# Evaluation of a transverse perineal approach to the canine vagina and vestibule

**DOI:** 10.3389/fvets.2024.1469961

**Published:** 2025-01-07

**Authors:** Sarah E. Saylor, Kyle G. Mathews

**Affiliations:** Department of Clinical Sciences, North Carolina State University College of Veterinary Medicine, Raleigh, NC, United States

**Keywords:** episiotomy, vaginal resection, urethrostomy, vaginal resection-anastomosis, canine, vagina, vestibule

## Abstract

There are a variety of surgical conditions impacting the canine vagina and vestibule that require access through a caudal approach. A standard vertical episiotomy involves making an incision beginning ventral to the anus and extending ventrally through the dorsal commissure of the vulva and into the lumen of the vestibule and distal vagina. The objective of this study was to determine if complex vaginal procedures could be performed via a transverse perineal approach, thus eliminating the need to incise and reconstruct the vulva as performed in a standard episiotomy, and to evaluate the feasibility of vaginal resection with vulvar-sparing vestibular urethrostomy using this transverse approach. Ten canine, female cadavers were obtained and randomly split into two equally sized groups. Cadavers in Group A (*n* = 5) had a vaginal resection-anastomosis cranial to the urethral papilla. Cadavers in Group B (*n* = 5) had a complete vaginectomy and vulvar sparing vestibular urethrostomy. Procedures for both groups were approached through a transverse perineal incision. Postoperatively, cadavers of both groups had right lateral pelvic radiographs taken with a calibration marker in the field. The distance between the location of the anastomosis (Group A) or vaginectomy site (Group B) and the skin incision was measured. The procedures in both Group A and Group B were successfully performed through a transverse perineal approach in all 10 cadavers. The mean transverse perineal incision length was 2.88 cm ± 0.49 cm, compared to a mean standard episiotomy length of 5.83 cm ± 0.79 cm. The mean distance between the location of the anastomosis (Group A) or vaginectomy site (Group B) and the skin incision measured on pre-contrast right lateral pelvic radiographs was 2.54 cm ± 0.34 cm. The results of this study confirm the feasibility of performing complex vaginal procedures through a transverse perineal approach. The described approach is shorter than the standard episiotomy, which may result in diminished discomfort and decreased surgical/anesthetic time. This approach also eliminates the need to reconstruct the vulva. Further evaluation in clinical patients is warranted.

## 1 Introduction

Surgical conditions impacting the canine vagina and vestibule that are accessible through an episiotomy include congenital abnormalities such as hydrocolpos, bands, septa, strictures, and stenoses, hormone dependent conditions such as vaginal edema, along with conditions more common in the older population such as neoplasia (1–3). Congenital abnormalities are a sequela to failed embryological development and are typically located just cranial to the urethral papilla. Commonly reported clinical manifestations of bands, septa, stenoses, and strictures include failed breeding, vaginal discharge, chronic urinary tract infections, and chronic vaginitis (1, 4–6). Hydrocolpos is an uncommon congenital condition in which vaginal distention occurs due to an obstruction and secondary accumulation of vaginal and uterine secretions (2, 7). The source of the obstruction in these cases included segmental aplasia of the vaginal mucosa and imperforate hymen (2, 7). Surgical correction of vaginal septa and strictures may involve excision and closure of the mucosa at the site of the lesion cranial to the urethral papilla. Vertical bands may be amenable to simple transection. Vaginal resection-anastomosis cranial to the urethral papilla is the recommended treatment for vaginal stenosis as opposed to T-shaped vaginoplasty or manual dilation (5, 6). The surgical procedure recommended for treatment of hydrocolpos is largely dependent on the source of the obstruction. Two cases of segmental vaginal mucosal aplasia were successfully treated with vaginal resection-anastomosis, while a case of imperforate hymen was able to be digitally broken down followed by endoscopic excision of residual tissue (2, 7). Vaginal edema is a condition in which bitches under the influence of estrogen, during proestrus or estrus, develop an edematous fold of mucosa originating from the ventral floor of the vagina, which can protrude through the vulvar opening, leading to mucosal irritation and ulceration. This condition is typically self-limiting and will resolve following the bitch’s heat cycle. In cases in which trauma to the tissue leads to irreparable tissue damage, surgical excision of the edematous tissue is recommended (3). An episiotomy is used in these cases to provide appropriate exposure for surgical correction. The conventional caudal episiotomy approach requires making an incision beginning just ventral to the anus and extending ventrally through the dorsal commissure of the vulva and into the lumen of the vestibule and distal vagina (3).

Neoplasia of the vagina and vestibule has a reported incidence of 2–3% of total canine neoplasms (8, 9). Seventy-three percent of vulvar and vaginal neoplasms have been reported to be benign, with 83% of these benign neoplasms being leiomyomas (8). Leiomyomas are more likely to occur in intact, nulliparous bitches (8–11). Benign neoplasms are commonly pedunculated, while malignant neoplasms are more likely to be broad-based (8). While pedunculated lesions are likely amenable to marginal excision via a caudal episiotomy, vulvovaginectomy, subtotal vaginectomy, or total vaginectomy in combination with an episiotomy for visualization are often performed for removal of broad-based tumors (1, 12). Vulvovaginal resection with perineal urethrostomy through a fusiform skin incision around the vulva and vulvovaginal resection with neo-urethrostomy in the floor of the vagina via a standard episiotomy approach have been described (13, 14). Vulvar-sparing vaginal resection/urethrostomy has not been widely described in the veterinary literature (10). In one study, an unspecified number of dogs had this procedure performed using a standard vertical episiotomy (10). A potential advantage of the vulvar-sparing technique is decreased exposure of the urethral stoma to fecal contamination.

The objective of this study was to investigate an alternative approach to the vagina and vestibule through a transverse perineal incision (TPI) that could be utilized for cases of vaginal pathology such as strictures, stenosis, and neoplasia impacting the vaginal vault, urethral papilla, or cranial vestibule, and to evaluate the feasibility of vaginal resection with vulvar-sparing vestibular urethrostomy using this transverse approach. A similar transverse approach has been described for surgical correction of rectovaginal fistulas in dogs but to our knowledge has not been described as an approach for more advanced surgical procedures of the vagina (15). If complex vaginal procedures could be performed via a transverse perineal approach, this may eliminate the need to incise and reconstruct the vulva as performed in a standard episiotomy. We hypothesized that a short TPI would allow adequate exposure for vaginal resection-anastomosis cranial to the urethral papilla and for complete vaginectomy with vulvar sparing vestibular urethrostomy.

## 2 Materials and methods

A total of 10 female canine cadavers were obtained. Cadavers were acquired from a local animal shelter following euthanasia. The dogs were euthanized for reasons unrelated to the study. Procedures were performed within 48 h of euthanasia for all cases. All cadavers were weighed pre-operatively. The cadavers were randomly assigned by coin toss to two, equally sized groups. Cadavers in Group A (*n* = 5) had a vaginal resection-anastomosis cranial to the urethral papilla. Cadavers in Group B (*n* = 5) had a midline celiotomy performed to either free the uterine stump in ovariohysterectomized females or transect the uterus to simulate an ovariohysterectomy, followed by a complete vaginectomy and vulvar sparing vestibular urethrostomy. The vaginal procedures for both groups were approached through a transverse perineal incision.

### 2.1 Description of transverse perineal incision approach

**Figure 1:**
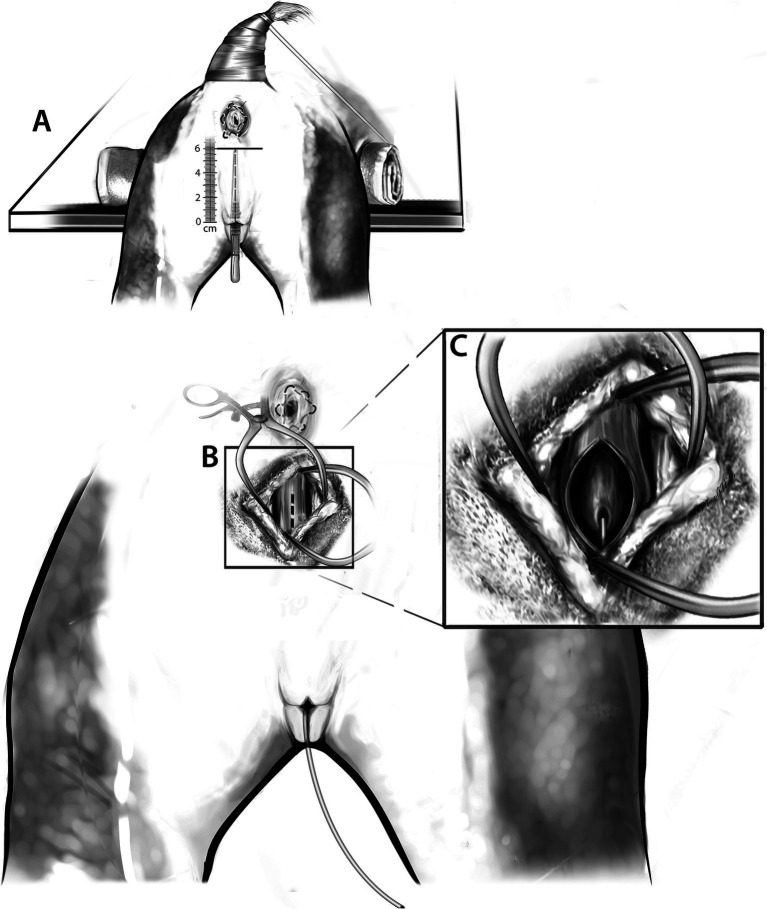
**(A)** The patient is positioned in sternal recumbency with the pelvic limbs positioned off the operating table. The pelvis is cushioned with a towel. The tail is reflected cranially, and an anal purse string suture is placed. For incisional planning, a scalpel handle is inserted into the vulva to determine the dorsal border of a standard vertical episiotomy incision. The length of the standard episiotomy is then measured. The horizontal black line is representative of the planned transverse skin incision. **(B)** The transverse perineal incision has been made, and subcutaneous tissues dissected. Gelpi retractors are placed to aid in visualization of the dorsal vaginal wall. The dotted line indicates the planned incision into the dorsal vagina. **(C)** Following incision into the dorsal vaginal wall, the urethral papilla and urinary catheter are visualized.

Cadavers were placed in sternal recumbency with the rear elevated and the hind limbs hanging off the table. The tail was taped forward, and a purse string suture was placed in the anus. A scalpel handle was inserted into the vestibule and used to determine the dorsal border of a standard vertical episiotomy incision, and a sharpie mark was made at this location. The vertical distance between this mark and the dorsal commissure of the vulva was measured. A transverse skin incision measuring one-half the length of the vertical distance was made at the level of the mark (dorsal extent of the standard episiotomy). If more exposure was required, this transverse incision was extended, and the final length of the incision recorded. Cadaver positioning and planning of the transverse perineal incision is illustrated in Figure 1A.

### 2.2 Description of vaginal resection-anastomosis (Group A)

**Figure 2:**
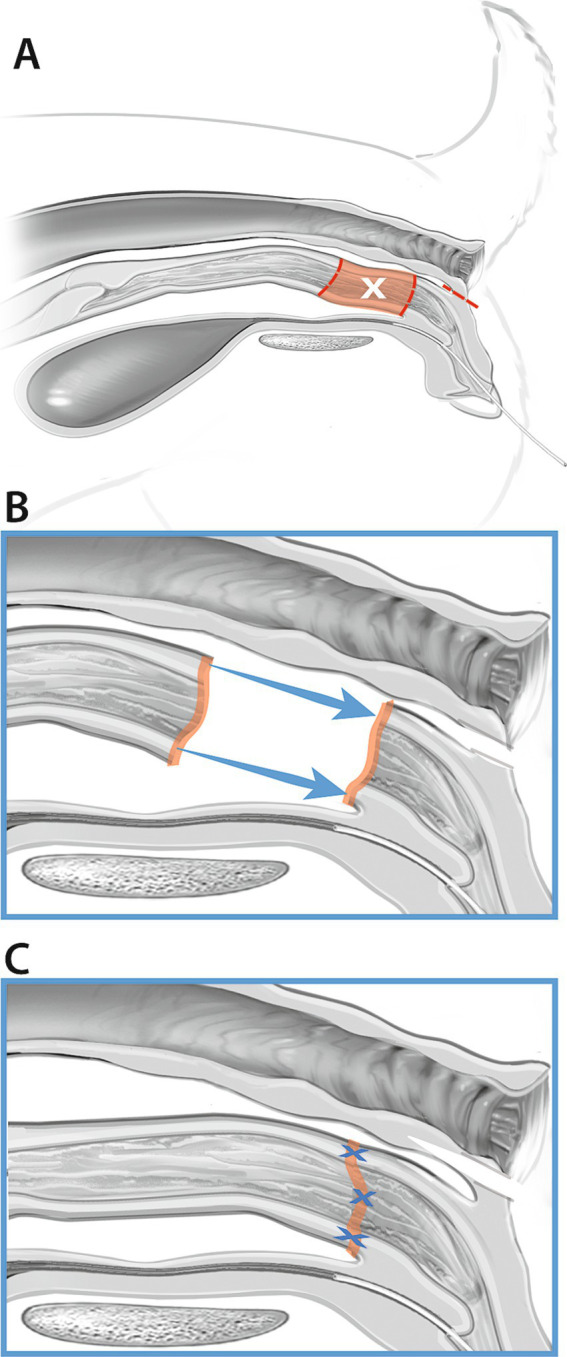
Vaginal resection and anastomosis (Group A). The dotted line between the anus and the vagina **(A)** represents dissection through the tissues between the rectum and the dorsal vaginal wall. The shaded region of the vagina in **(A)** represents the 2 cm segment of vagina that was resected. The vagina was then apposed using 3-0 PDS **(B,C)**.

Either an 8Fr or 10Fr foley catheter was placed into the urethra of all cadavers either prior to the initial skin incision or following incision into the vagina, which allowed direct visualization of the urethral papilla. Following the transverse skin incision, a combination of sharp and blunt dissection with Metzenbaum scissors and monopolar cautery was used to longitudinally dissect through the retractor clitoridis muscles, constrictor vestibuli muscle, and associated subcutaneous tissues. A longitudinal incision was made into the dorsal wall of the vagina using monopolar cautery. The urethral papilla was visualized. An incision into the dorsal wall of the vagina and visualization of the urethral papilla with a urinary catheter in place is illustrated in Figures 1B,C. Blunt dissection was used to free the serosa of the vagina circumferentially 3 cm cranial to the urethral papilla. A circumferential incision was made into the vagina with cautery just cranial to the urethral papilla, meeting the cranial edge of the initial vaginal incision dorsally. Allis tissue forceps were used to grasp the dorsal and ventral margins of the vagina to aid in caudal retraction. A second circumferential vaginal incision was made 2 cm cranially. The incised ring of vaginal tissue was removed. A stay suture was placed in the dorsal wall of the remaining cranial vagina. The urethral papilla was marked with a single skin staple. Re-apposition of the vagina was achieved with simple interrupted sutures using 3-0 PDS, starting ventrally and working up the lateral walls. The dorsal vaginal wall was closed in combination with the initial longitudinal incision. The anastomotic site was marked with 2 skin staples laterally. The muscular layers and subcutaneous tissue were closed with 3-0 PDS in a cruciate pattern, and staples were used to appose the skin edges. Figure 2 should be referenced for a visual representation of the vaginal resection-anastomosis procedure.

### 2.3 Description of complete vaginectomy and vulvar sparing vestibular urethrostomy (Group B)

#### 2.3.1 Midline celiotomy

Each cadaver was placed in dorsal recumbency. A skin incision was made using a scalpel blade from just cranial to the umbilicus to the pubis. Sharp dissection was continued with the scalpel blade through the subcutaneous tissues. A stab incision was made into the linea alba and extended caudally with the scalpel. The bladder was reflected ventrally out of the body to allow exposure of the uterus. A combination of sharp and blunt dissection was used to break down the attachments of the bladder to the vagina forming the vesicogenital pouch, and the same was done for the attachments of the rectum to the vagina forming the rectogenital pouch. The vaginal and uterine arteries were ligated and transected. Care was taken to preserve the caudal vesical artery supplying the bladder. If the dog was intact, two transfixing ligatures were placed in the uterine body just cranial to the cervix using 0 PDS, and the uterus was transected to simulate an ovariohysterectomy. A stay suture was placed in the uterine stump using 3-0 PDS to aid in location during the vaginectomy procedure. The body wall was closed using 0 PDS in a simple continuous pattern.

#### 2.3.2 Vaginectomy and urethrostomy

**Figure 3:**
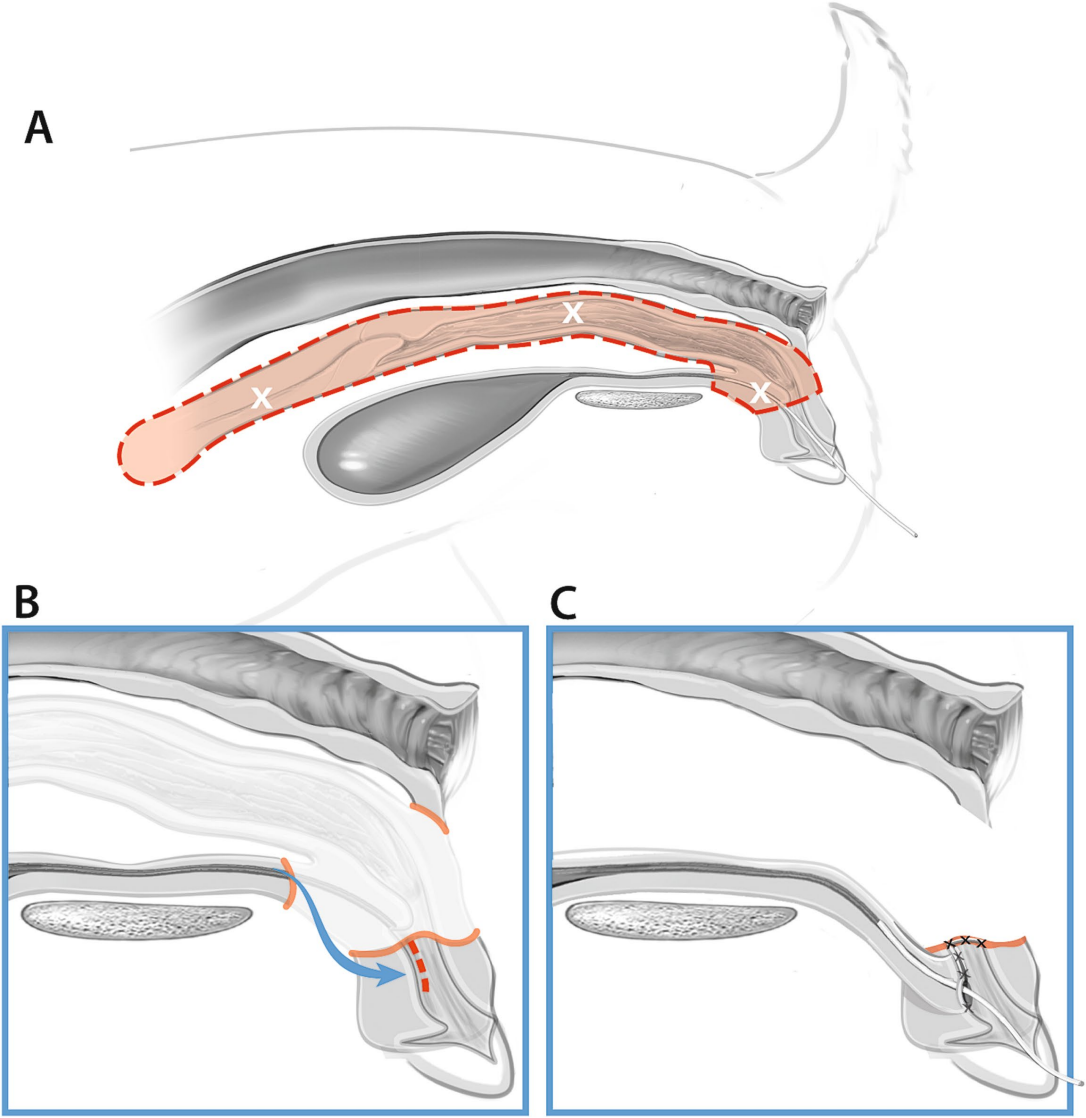
Vaginectomy and urethrostomy procedure (Group B). **(A)** The red dotted line and shaded area illustrates the uterus, vagina, and segment of urethra removed during the procedure. **(B)** The remaining distal urethra is moved caudally to the floor of the vestibule. **(C)** The distal urethra is catheterized and sutured to the floor of the vestibule and the vestibule is sutured closed just cranial to the urethrostomy.

Either an 8Fr or 10Fr foley catheter was placed into the urethra of all cadavers either prior to the initial skin incision or following incision into the vagina, which allowed direct visualization of the urethral papilla. Following the transverse skin incision, a combination of sharp and blunt dissection with Metzenbaum scissors and monopolar cautery were used to longitudinally dissect through the retractor clitoridis muscles and constrictor vestibuli muscles. Monopolar cautery was used to make a longitudinal incision into the dorsal vaginal wall. The urethral papilla and urinary catheter were visualized (Figure 1A). A combination of sharp and blunt dissection was used to free the lateral and dorsal border of the vagina cranially until the uterine stump was able to be located and reflected caudally. The ventral vagina was freed to the level of urethral insertion. The uterine stump was then placed back into its normal anatomical location within the pelvis. A “V” shaped incision was made into the lateral walls and floor of the vestibule just caudal to the urethral papilla at the vestibulovaginal junction. Metzenbaum scissors were used to free any residual ventral attachments of the vagina to surrounding subcutaneous tissues. The urethra was sharply transected 2 cm cranial to the papilla. Once freed of all residual attachments, the vagina and associated 2 cm of urethra were removed. A stay suture was placed into the dorsal border of the remaining urethra with 3-0 PDS. The urethra was moved caudally, and 3-0 PDS in a simple interrupted pattern was used to attach the urethra to the mucosal surface of the floor of the vestibule at the previously made incision. A skin staple was placed to mark the location of the urethrostomy in the vestibule. The lateral walls of the vestibule were sutured together and to the dorsal aspect of the urethra in a simple interrupted pattern using 3-0 PDS. The dorsal aspect of the vestibule was closed with cruciate sutures using 3-0 PDS. The retractor clitoridis muscles were apposed using 3-0 PDS. Staples were placed to appose the skin. Figure 3 should be referenced for a visual representation for the vaginectomy and urethrostomy procedure.

In one cadaver, the urethra was noted to be iatrogenically transected when the uterine stump was reflected. Transection likely occurred during dissection within the abdomen. In this dog, 3.5 cm of urethra was removed. The urethrostomy was still able to be performed despite the longer length of urethra removed, and the procedure was completed as described above.

### 2.4 Imaging

**Figure 4:**
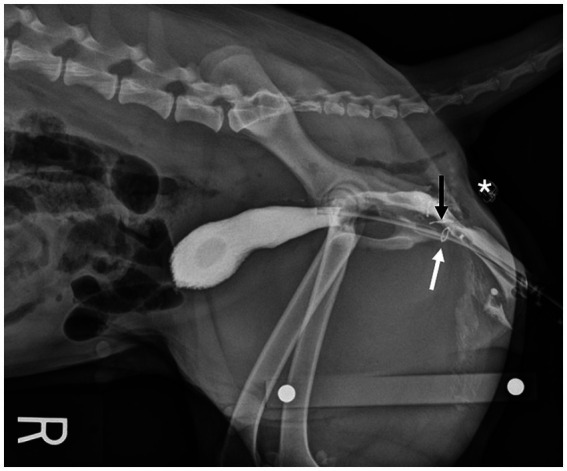
Lateral radiograph of a Group A cadaver. Staples placed at the urethral papilla (white arrow) and vaginal anastomosis site (black arrow) orient the viewer to the location of the urethra in respect to the anastomosis. Location of the transverse skin incision (*).

Immediately postoperatively, cadavers of both groups had right lateral pelvic radiographs taken with a calibration marker in the field. The distance between the location of the anastomosis (Group A) or vaginectomy site (Group B) and the skin incision was measured. In Group A, positive contrast cystourethrography was performed to give detail regarding location of the bladder and urethra in comparison to the vaginal anastomosis site (Figure 4). To perform the cystourethrogram, iodinated contrast was injected into the bladder (Omnipaque, iohexol 240 mgI/mL), and a right lateral radiograph was taken.

**Figure 5:**
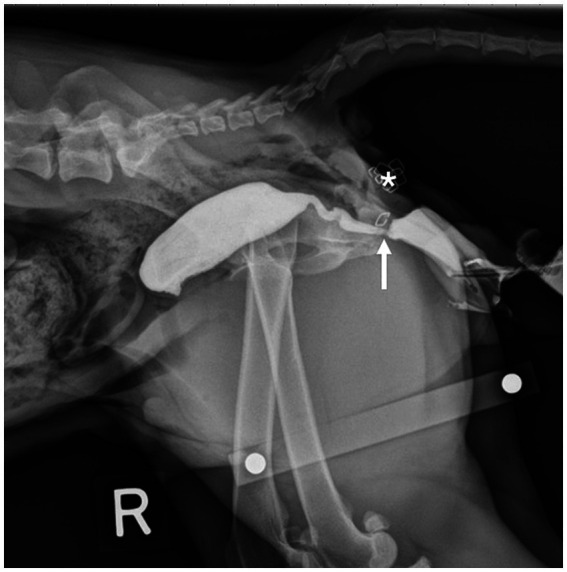
Lateral radiograph of a Group B cadaver. A staple placed at the urethrostomy site can be seen to orient the viewer to the location of the caudal vestibular urethrostomy (arrow). Location of the transverse skin incision (*).

In Group B, positive contrast cystourethrography and positive contrast vaginography (Omnipaque, iohexol 240 mgI/mL) were performed to highlight the location of the vaginectomy and urethrostomy (Figure 5). Prior to injection of contrast media, the labia were sutured closed around the urinary catheter using 0 PDS in a simple continuous pattern to minimize contrast leakage. Following cystourethrogram, a vaginogram was performed after deflating the urinary catheter balloon and withdrawing the catheter into the vestibule while contrast was continually instilled. A right lateral radiograph was then taken.

## 3 Results

The mean weight of cadavers used in this study was 20.37 kg ± 6.03 kg. The estimated age was 1–2 years. Both the vaginal resection-anastomosis procedure and the complete vaginectomy with vulvar sparing vestibular urethrostomy procedures were successfully performed in all cadavers through a TPI approach. The mean transverse perineal incision length was 2.88 cm ± 0.49 cm, compared to a mean standard episiotomy length of 5.83 cm ± 0.79 cm. In two cadavers, the TPI length was increased to improve exposure. In these cadavers, TPI lengths were 2.5 cm and 3 cm shorter than the standard episiotomy length. The TPI length was a mean of 2.95 ± 0.48 cm shorter than the measured standard episiotomy length, and the TPI was a minimum of 2.50 cm shorter than the standard episiotomy length in all cadavers. The mean distance between the location of the anastomosis (Group A) or vaginectomy site (Group B) and the skin incision measured on pre-contrast right lateral pelvic radiographs was 2.54 cm ± 0.34 cm. One intraprocedural complication was encountered in Group B. Transection of the urethra was suspected to have occurred during abdominal dissection in one cadaver. In this cadaver, 3.5 cm of urethra was removed along with the vagina, and vestibular urethrostomy was successfully completed. No intraprocedural complications were encountered in Group A. The caudal vestibular urethrostomy was able to be performed without complication through the described transverse perineal approach.

## 4 Discussion

The results of this study confirm the feasibility of performing vaginal resection anastomosis and complete vaginectomy with vulvar sparing vestibular urethrostomy through the described transverse perineal approach. The mean TPI was 2.95 cm shorter than the mean standard episiotomy length, and it eliminated the need to reconstruct the vulva in multiple layers. The shorter incision needed for a transverse perineal approach and the elimination of an episiotomy may decrease post-operative pain and surgical/anesthetic time. Urinary tract infection and urine scald are complications that have been described following vulvovaginectomy and perineal urethrostomy (14). The vestibular sparing urethrostomy described in this study may minimize fecal contamination and secondary urinary tract infections by maintaining vulvar coverage of the urethrostomy site.

This study has several limitations. Intra-operative complications such as hemorrhage could not be assessed. Similarly, post-operative complications and outcomes could not be evaluated. The described complication in one Group B cadaver would likely have been avoided if urethral catheterization was performed prior to midline celiotomy to aid in intra-operative identification of the pelvic urethra. We do not believe that this complication was related to the TPI approach. A prospective study comparing a standard episiotomy to the TPI approach will be necessary to determine whether the TPI decreases morbidity, including pain, in clinical patients. Additional investigation in clinical patients is also necessary to determine whether the vestibular sparing urethrostomy decreases the risk of secondary urinary tract infection and urine scald. In conclusion, the transverse perineal approach is a promising alternative to the standard episiotomy, and it provides the surgeon with adequate exposure for surgical treatment of complex conditions of the vagina and vestibule.

## Data Availability

The raw data supporting the conclusions of this article will be made available by the authors, without undue reservation.
